# Case Report: Ofatumumab for the treatment of refractory anti-NMDAR-positive autoimmune encephalitis

**DOI:** 10.3389/fimmu.2026.1754979

**Published:** 2026-06-10

**Authors:** Yekai Ma, Yani Tang, Qinyue Peng, Weiqing Zhang, Yongjun Wu, Jun Chen, Susu Xiong, Dong Lv

**Affiliations:** 1 Department of Psychiatry and Psychology, Affiliated Hospital of Guangdong Medical University, Zhanjiang, China; 2 Radiology Imaging Centre, Affiliated Hospital of Guangdong Medical University, Zhanjiang, China; 3 Department of Neurology, Affiliated Hospital of Guangdong Medical University, Zhanjiang, China

**Keywords:** anti-NMDAR encephalitis, CD20 monoclonal antibody, immunotherapy, ofatumumab, refractory

## Abstract

This study reports three cases of refractory anti-NMDAR encephalitis patients who exhibited persistent severe neuropsychiatric symptoms after first-line immunotherapy (glucocorticoids, intravenous Immunoglobulin) and long-term (maintenance) immunotherapy (mycophenolate mofetil). These patients received subcutaneous injections of ofatumumab (OFA, 20 mg per dose), with dynamic monitoring of CD20+ B cell levels, antibody titers, imaging findings, and neurological function scores. All three patients showed significant improvement in psychiatric symptoms, cognitive function, and brain imaging after treatment, with CD20+ B cell levels rapidly declining to extremely low levels. Only one patient experienced mild bone pain and a low-grade fever, with no severe adverse events reported. Therefore, OFA provides a rapid, safe, and effective approach to alleviate symptoms in refractory anti-NMDAR encephalitis, offering a promising and well-tolerated therapeutic option for patients unresponsive to conventional treatments.

## Introduction

Anti-N-methyl-D-aspartate receptor (NMDAR) encephalitis is one of the most common types of autoimmune encephalitis (AE), first systematically described in 2007 by Dalmau et al. (1). The disease is mediated by autoantibodies targeting the NMDAR GluN1 subunit, leading to receptor cross-linking and internalization, which results in synaptic dysfunction and neuropsychiatric symptoms (2). Typical clinical manifestations include cognitive impairment, psychiatric and behavioral abnormalities, epileptic seizures, involuntary movements, language disorders, and decreased level of consciousness (3).

Currently, the standard immunotherapy strategies for anti-NMDAR encephalitis are divided into first-line and second-line treatments. First-line immunotherapy includes high-dose glucocorticoid pulse therapy, intravenous immunoglobulin (IVIG), and plasma exchange (PE) (4). For patients with poor response to first-line treatment, both domestic and international guidelines recommend early initiation of second-line immunotherapy, including rituximab (RTX), cyclophosphamide, and mycophenolate mofetil (5–7). RTX, as a first-generation chimeric anti-CD20 monoclonal antibody, effectively eliminates B cells and has demonstrated favorable efficacy in multiple studies (8, 9). However, RTX carries risks such as infusion reactions, serum sickness, severe infections, and rare cases of progressive multifocal leukoencephalopathy (10), making it unsuitable for some patients due to allergic reactions or concerns.

Ofatumumab (OFA) is a second-generation fully humanized anti-CD20 monoclonal antibody administered via subcutaneous injection and has been approved for the treatment of relapsing multiple sclerosis (11, 12). Compared to RTX, OFA offers advantages such as lower immunogenicity, fewer infusion-related reactions, and convenient administration (5). In recent years, OFA has been explored for the treatment of autoimmune encephalitis. Preliminary case series demonstrate its efficacy in refractory or recurrent anti-NMDAR encephalitis (13, 14).In a prospective, multicenter cohort study published by Guo in 2025, 93.1% (54/58) of patients showed further improvement in the mRS score after treatment, with a median improvement time of 14 days (15).

However, current real-world data on OFA treatment for anti-NMDAR encephalitis remain extremely limited, particularly with scarce clinical experience in the Chinese population. Published literature predominantly consists of case reports or small-sample retrospective analyses, lacking dynamic, multidimensional quantitative evaluations. This study presents three cases of anti-NMDAR encephalitis refractory to first-line and/or second-line immunotherapy, in which OFA was initiated based on its potential therapeutic benefit. All three patients demonstrated rapid clinical improvement, with CD20^+^ B-cell counts falling to near-undetectable levels. They regained the ability to perform daily activities and showed marked recovery of cognitive function. Follow-up Electroencephalogram (EEG), cranial magnetic resonance imaging (MRI), and MMSE scores all confirmed effective and sustained disease control. We described these three patients, all exhibiting prominent psychotic symptoms in detail, monitored multidimensional indicators, including B-cell levels, antibody titers, imaging findings, and neurological function scales (MMSE, CASE, mRS), dynamically, and evaluated the efficacy, safety, and dosing regimen feasibility of OFA usage, aiming to provide real-world evidence of personalized second-line immunotherapy strategies for clinical practice.

## Case reports

### Case 1

A 15-year-old female was admitted with a one-year history of right upper limb convulsions accompanied by psychiatric disturbance. The patient experienced her first episode in early November 2022, presenting with right upper limb convulsions, impaired consciousness, oral hypersalivation, trismus, perioral cyanosis, and behavioral abnormalities. She had no prior history of epilepsy or psychiatric disorders, and no antecedent fever or other psychological stressors. Lumbar puncture revealed a CSF anti-NMDAR antibody titer of 1:1 with negative serum NMDAR antibodies. Dynamic EEG showed moderate diffuse abnormalities, and brain MRI demonstrated cerebral atrophy. A diagnosis of anti-NMDAR autoimmune encephalitis was established. Treatment included methylprednisolone, intravenous immunoglobulin (IVIG) bolus therapy, and neurology intensive care, which controlled epileptic symptoms. However, post-discharge maintenance therapy with oral corticosteroids, antiepileptic drugs, and mycophenolate mofetil failed to achieve complete symptom remission, leaving residual psychiatric symptoms and EEG abnormalities.

In March 2024, the patient was readmitted due to aggravated psychiatric symptoms, emotional irritability, and shouting. Follow-up lumbar puncture revealed serum anti-NMDAR antibody titers of 1:1, CSF titer of 1:10, oligoclonal zone band type II, IgG index of 0.55, 24-hour intrathecal IgG synthesis rate of 1.39 mg/24 h, and serum CD20^+^ B cell count of 156.38 cells/μL. Brain MRI showed cerebral atrophy, while pelvic MRI revealed no abnormalities. Physical examination revealed clear consciousness, a moon face, a buffalo hump, and facial flushing. Neurological examination identified no abnormalities except cognitive impairment, with a Mini-Mental State Examination (MMSE) score of 24. After multidisciplinary consultation, the patient was considered for treatment with corticosteroids, gamma globulin, and mycophenolate mofetil, but clinical recovery was not achieved due to persistent psychiatric abnormalities, cognitive impairment, EEG abnormalities, intermittent emotional instability, and convulsive episodes, along with frequent adverse effects of corticosteroids. After thorough communication with the patient and family members and obtaining informed consent, the B-cell depletion agent OFA was administered. The first OFA dose of 20 mg improved convulsions, delirium, and cognitive impairment, with a significant decrease in serum CD20+B cell count to 0.24 cells/μL. A transient low-grade fever and bone pain occurred on the injection day, which resolved with symptomatic treatment. Subsequent OFA subcutaneous injections were administered in May, August, and December 2024, with follow-up B-cell counts consistently remaining within low ranges. Follow-up EEG showed normal results, and the MMSE score remained at 28. During the follow-up visit 2 years after discharge, the patient showed no convulsions, cognitive impairment, or psychiatric abnormalities, with no impact on daily activities and a favorable prognosis.

### Case 2

A 21-year-old male was admitted in June 2024 due to headache and convulsions. He had no history of epilepsy, psychiatric disorders, fever, or other psychological stressors. Neurological examination revealed no abnormalities. CSF tests showed anti-NMDAR and anti-MOG antibody titers of 1:10. CSF analysis: white blood cells 60×10^6^/L, total protein 764.40 mg/L, and microalbumin 598.80 mg/L. Head MRI indicated localized gyral abnormal signals in the left temporal lobe with mild swelling. Enhanced scanning revealed slightly increased vascular density and thickening in the left temporal-parietal region. Electroencephalogram (EEG) showed widespread mild abnormal activity. The diagnosis was considered to be acute encephalopathy (AE). Treatment included intravenous methylprednisolone pulse therapy and intravenous immunoglobulin (IVIG) (0.4 g/kg/day for 5 days), along with antiepileptic therapy. The patient’s condition improved without headache or seizures. Oral corticosteroids were administered, but after 1 week, auditory hallucinations, visual hallucinations, persecutory delusions, attention deficits, memory impairment, and right upper limb involuntary movements occurred. Follow-up lumbar puncture revealed CSF white blood cells 20×10^6^/L, total protein 576.20 mg/L, and microalbumin 318.70 mg/L. Serum and CSF anti-NMDAR (+) 1:10, anti-MOG (+) 1:32, oligoclonal band type I, IgG index 0.84, and 24-hour intrathecal IgG synthesis rate 22.89 mg/24 h. Reproductive system ultrasound showed no tumors. Dynamic EEG remained normal. Despite corticosteroid and symptomatic treatment, symptoms did not improve. The absolute count of CD20^+^ B cells was 738.89 cells/μL. OFA therapy was initiated after comprehensive evaluation. No significant adverse reactions were observed, with CD20^+^ B cell levels decreasing to low levels, rapid improvement in cognitive function, absence of hallucinations or delusions, no involuntary movements, and resolution of the original lesion on head MRI. Four months after discharge, OFA therapy was repeated. and multiple follow-up tests showed consistently low CD20^+^ B cell counts. During a 2-year post-discharge follow-up, the patient remained asymptomatic and maintained normal daily life and work activities.

### Case 3

A 39-year-old male with a confirmed diagnosis of anti-NMDAR encephalitis had been hospitalized three times within a period of over one year. Prior to onset, there was no history of epilepsy or psychiatric disorders. The main symptoms during previous hospitalizations included convulsions, fever, blurred vision, and impaired consciousness. Treatment involved intravenous methylprednisolone pulse therapy and intravenous immunoglobulin (IVIG) (0.4 g/kg/day for 5 days), which alleviated symptoms. Subsequent maintenance therapy included oral corticosteroids and mycophenolate mofetil. Final anti-NMDAR autoimmune encephalitis antibody tests revealed a serum titer of 1:10 and a cerebrospinal fluid (CSF) titer of 1:3.2.

In July 2025, the patient was readmitted due to sudden-onset of consciousness loss, delirious speech, and emotional agitation. There was no history of antecedent fever or other psychological stressors. The patient exhibited delirium and significant impairment of cognitive function, prominent visual hallucinations, and persecutory delusions. Neurological examination was otherwise unremarkable. Admission assessments yielded the following scores: MMSE 8, CASE 5, and mRS 3. CSF results showed a leukocyte count of 30×10^6^/L, oligoclonal band type 2, with oligoclonal bands detectable in the CSF; IgG 24-hour synthesis rate was 8.42 mg/24 h, and anti-NMDAR antibody titers in both serum and CSF were 1:10. Brain MRI revealed new patchy abnormal signals in the right temporal lobe and shallowing of the right temporal sulcus, while PET-CT showed no tumors. Intravenous methylprednisolone pulse therapy and intravenous immunoglobulin (IVIG) (0.4 g/kg/day for 5 days) were administered; however, cognitive impairment and agitation symptoms showed no significant improvement, and ambulatory EEG revealed widespread mild abnormal activity. Given the poor response to first-line immunotherapy, OFA treatment was initiated. OFA was administered on days 1, 7, and 14, with CD20^+^ B cell levels remaining consistently low. The patient experienced no adverse reactions. After three doses, psychiatric symptoms markedly improved, with stabilized mood, absence of delirious speech or impulsivity, and significant enhancement in calculation and memory abilities. Follow-up lumbar puncture revealed CSF leukocyte count of 3×10^6^/L, oligoclonal band type 1, IgG 24-hour synthesis rate of 8.46 mg/24h, and reduced CSF antibody titer to 1:3.2. The follow-up head MRI after the first monoclonal antibody treatment showed a slight reduction in the lesion size in the right temporal lobe compared to previous findings. Discharge assessment scores were as follows: MMSE 22, CASE 2, and mRS 1.

The patient returned for follow-up one month after discharge with an MMSE score of 26, a CASE scale score of 1, and an mRS score of 1. CD20+ B cells remained at low levels, and head MRI indicated further reduction of the right temporal lobe lesion without significant viral or bacterial infection. The patient’s functional status showed improvement compared to baseline. At one-year follow-up after discharge, no symptom recurrence was observed, and daily activities remained normal. **Figure 1** illustrates the changes in various scales before and after treatment in three patients. **Figure 2** shows the lesion changes before and after treatment in Case 2 and Case 3. **Figure 3** monitors the fluctuations in CD20+ B cell counts throughout the disease course of all three patients. **Table 1** presents the demographic, clinical, and immunological characteristics of three patients.

**Figure 1:**
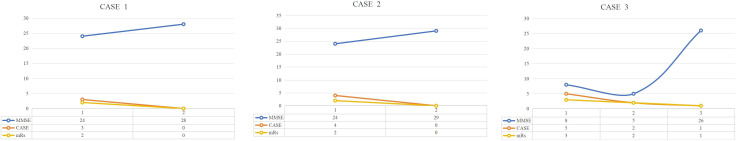
Changes in hospitalization scale scores across the three cases. MMSE, Mini-Mental State Examination; mRS, Modified Rankin Scale; CASE, Clinical Assessment Scale for Autoimmune Encephalitis.

**Figure 2:**
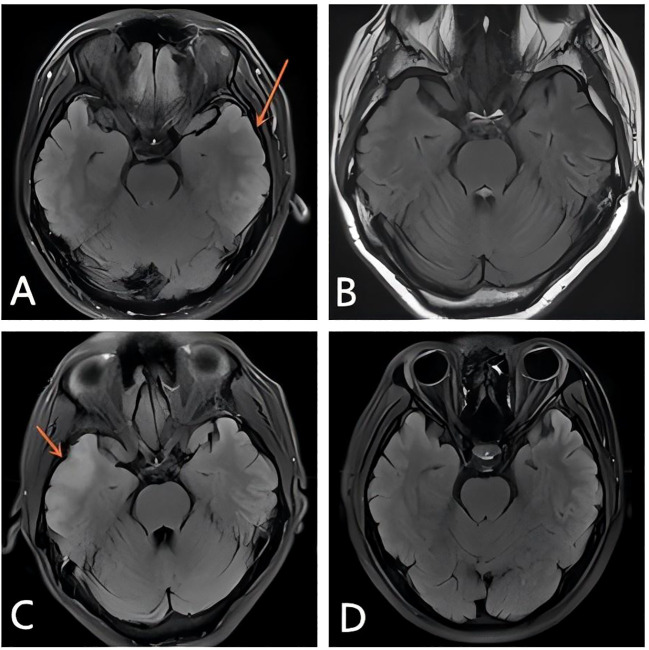
Changes in T2-weighted liquid-attenuated inversion recovery (LAIR) MRI before and after OFA treatment: **(A)** Case 2 shows focal abnormal signal in the left temporal lobe with mild swelling; **(B)** Case 2 demonstrates normalization of the lesion following OFA treatment; (**(C)** Case 3: Patchy abnormal signal on the right temporal lobe; **(D)** Case 3: Reduction in lesion size following OFA treatment.

**Figure 3:**
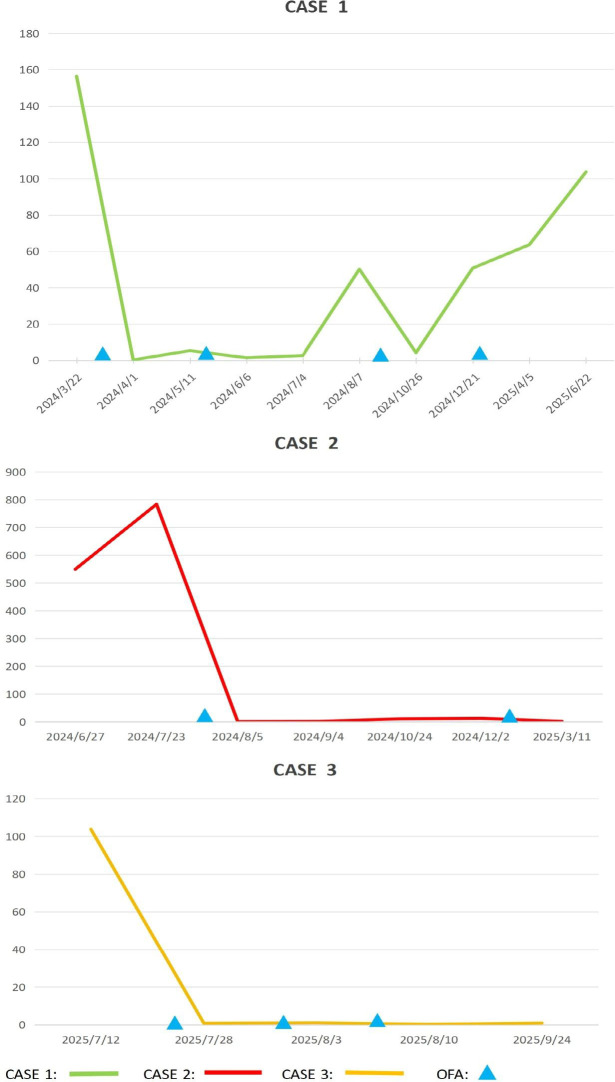
Changes in CD20+ B-cell counts during treatment in three patients. OFA, ofatumumab.

**Table 1:** Demographic, clinical, and immunological characteristics of three patients with refractory anti-NMDAR encephalitis treated with ofatumumab.

Patient	1 (15/F)	2 (21/M)	3 (39/M)
Demographics and baseline status			
Disease duration prior to OFA, mo	16	<1	>12 (multiple relapses)
Prior immunotherapies	IVIG 0.4 mg/kg × 5 days; MP 1 g decreasing; MMF	IVIG 0.4 mg/kg × 5 days; MP 1 g decreasing	IVIG 0.4 mg/kg × 5 days; MP 1 g decreasing; MMF
Concomitant autoantibodies	—	MOG (serum and CSF)	—
Pre-OFA clinical and paraclinical findings			
Predominant refractory manifestations	Persistent psychiatric symptoms, cognitive deficits, and electrographic abnormalities	Visual/auditory hallucinations, persecutory delusions, involuntary movements (right upper limb)	Impaired consciousness, incoherent speech, agitation, prominent psychosis
Serum/CSF anti-NMDAR antibody titer	1:1/1:10	1:10/1:10	1:10/1:10
CSF leukocyte count,/μL	1	20	30
CSF total protein, mg/L	276.1	576.2	626.7
CSF oligoclonal bands (type)	Type II	Type I	Type II
CSF IgG index	0.55	0.84	0.67
24-h intrathecal IgG synthesis rate, mg/24 h	1.39	22.89	8.42
Peripheral CD20^+^ B-cell count, cells/μL	156.38	738.89	103.68
Brain MRI findings	Cerebral atrophy	Left temporal cortical hyperintensity with mild edema (resolved on follow-up)	Right temporal subcortical/patchy hyperintensity with sulcal atrophy
Baseline EEG findings	Moderate abnormalities	Mild generalized abnormalities	Widespread mild abnormalities
Baseline MMSE score	24	24	8
Baseline CASE score	3	4	3
Baseline mRS score	2	2	3
Ofatumumab administration			
Route and schedule	SC 20 mg ×4 doses (0, 2, 5, 9 mo)	SC 20 mg ×2 doses (0, 4 mo)	SC 20 mg ×3 doses days 1, 7, and 14
Post-OFA outcomes and follow-up			
Nadir peripheral CD20^+^ B-cell count, cells/μL	0.24	0.57	0.74
Follow-up CSF anti-NMDAR antibody titer	1:10	1:10	1:3.2
Follow-up CSF leukocyte count,/μL	6	1	3
Follow-up oligoclonal bands (type)	Type II	Type I	Type I
Follow-up brain MRI	Cerebral atrophy (stable)	Normalized	Reduction in extent of right temporal lesion
Follow-up EEG	Normal	Normal	—
Latest MMSE score	28	29	26
Latest CASE score	0	0	1
Latest mRS score	0	0	1
Clinical response	Seizure-free; full cognitive and psychiatric recovery	Complete resolution of hallucinations, delusions, and involuntary movements	Marked improvement in agitation, speech coherence, and cognition
Adverse events attributed to OFA	Transient low-grade fever and bone pain post-injection	—	—

CSF, cerebrospinal fluid; EEG, electroencephalography; IgG, immunoglobulin G; IVIG, intravenous immunoglobulin; MMF, mycophenolate mofetil; MP, methylprednisolone; MOG, myelin oligodendrocyte glycoprotein; mRS, modified Rankin Scale; MMSE, Mini-Mental State Examination; CASE, Clinical Assessment Scale for Autoimmune Encephalitis; NMDAR, N-methyl-d-aspartate receptor; OFA, ofatumumab; SC, subcutaneous.

*NMDAR autoantibodies in serum and cerebrospinal fluid samples were tested by an independent third-party medical laboratory (KingMed Diagnostics, Guangzhou) using the CBA method in accordance with laboratory-developed test standard.s.

## Discussion

The core pathological mechanism of anti-NMDAR encephalitis involves the binding of autoantibodies to the GluN1 subunit of NMDARs, leading to receptor cross-linking, internalization, and functional impairment, which subsequently results in impaired synaptic plasticity in the hippocampus and prefrontal cortex (1, 2). This receptor dysfunction explains the prominent cognitive deficits and psychiatric symptoms observed in patients. Although the majority of patients respond well to first-line immunotherapy, approximately 20%–30% of patients exhibit refractory disease, characterized by persistent or recurrent symptoms despite first-line and at least one second-line treatment (5, 16).

As a second-generation anti-CD20 monoclonal antibody, OFA exerts its therapeutic effects not only through complement-dependent cytotoxicity (CDC) and antibody-dependent cell-mediated cytotoxicity (ADCC), but also demonstrates enhanced CDC activity. This phenomenon may stem from the fact that the binding epitope of ofatumumab on CD20 is positioned closer to the plasma membrane, facilitating Fc-mediated complement binding near the cell surface. This characteristic may enhance the CDC efficacy induced by ofatumumab, enabling more efficient clearance of B cell subsets with lower CD20 expression levels (17). Additionally, OFA is a fully humanized antibody, exhibiting significantly lower immunogenicity compared to RTX (18). In ASCLEPIOS I and II trials, the incidence of infusion-related reactions to OFA was 20.2%, while the incidence of RTX was 42–78.3% (19, 20).The incidence of anti-drug antibodies against OFA was 0.2%, significantly lower than the 7% observed with RTX (19). Its subcutaneous administration eliminates the prolonged monitoring required for intravenous infusion, thereby improving patient compliance.

In this study, all three patients achieved significant clinical improvement after OFA treatment. CD20+ B cells rapidly declined to near-zero levels following the initial dose and remained at low levels during subsequent follow-ups, consistent with pharmacodynamic data from previous multiple sclerosis studies (12). At one-month follow-up, expect clinical symptoms improvements, we noticed that the CSF antibody titer decreased from 1:10 to 1:3.2, and the cranial MRI demonstrated progressive reduction of the right temporal lobe lesion, providing direct evidence of central inflammatory remission for case 3, while case 2 showed mainly MRI improvement, and there was no change in antibody titers or MRI changes for case 1, although the symptoms improved rapidly. No relapse was observed for long-term follow-up, respectively, in Case 1 (follow-up: 15 months), Case 2 (follow-up: 2 years), or Case 3 (follow-up: 1 year), indicating that OFA induces long-term immune remission. Compared with the published literature on OFA treatment for anti-NMDAR encephalitis, the therapeutic outcomes in this study were consistent with studies reported previously. Lai et al. reported three cases of severe refractory anti-NMDAR encephalitis patients who achieved complete recovery after OFA treatment. In one patient, the cerebrospinal fluid antibody titer decreased from 1:10 to 1:3.2, and Case 3 in the current study also exhibited a similar change (21). Gong et al. reported significant improvement in clinical symptoms and antibody titers in two patients following OFA therapy (13). A systematic review involving 15 patients demonstrated an overall efficacy rate of 87% (13/15) after OFA treatment, with the longest reported follow-up period being 82 weeks (14).

Notably, all three patients exhibited significant cognitive impairment while admitted, with a marked improvement in MMSE scores after OFA treatment (Case 1: 24→28; Case 3: 8→26). The mechanisms underlying the cognitive improvement may include: (1) The clearance of B cells significantly alleviates early-stage inflammation, reduces pathogenic antibodies, and mitigates damage to synaptic function; on the other hand, OFA regulates cytokines and influences microglial polarization, ultimately exerting a neuroprotective effect that reduces the sustained production of pathogenic antibodies; (2) After OFA depletes B cells, it modulates the cytokine network by reducing the Th17.1 cell subset and associated pro-inflammatory cytokines, thereby indirectly alleviating neuroinflammation; (3) It has been demonstrated that B cell clearance alters microglial polarization from the pro-inflammatory M1 phenotype to the neuroprotective M2 phenotype, thereby promoting synaptic repair and neural regeneration (22).

For medication safety assessment, only Case 1 exhibited transient low-grade fever and bone pain, both mild and self-limiting, with no severe infections or infusion-related adverse events. This aligns with the safety profile of OFA observed in large-scale clinical trials for multiple sclerosis, where the incidence of severe infections is approximately 2.5 per 100 participants per year (11).In the previously reported series on the use of OFA for treating AE, three patients experienced short-term low-grade fever without symptoms of respiratory or urinary tract infection (23). Nevertheless, the infection risk associated with long-term B-cell depletion (particularly progressive multifocal leukoencephalopathy and hepatitis B reactivation) warrants vigilance, and routine screening for latent infections such as hepatitis B and tuberculosis should be performed prior to treatment (24).

## Limitations and future directions

Limitations of this study: (1) The sample size was extremely small (only 3 cases), making statistical inference impossible; (2) It was a single-center retrospective study, which introduced selection bias; (3) The maximum follow-up duration was 2 years, insufficient to assess long-term recurrence rates and delayed adverse events; (4) The dosing regimens were not standardized, with varying injection frequencies and total doses across the 3 patients, precluding the determination of an optimal regimen; (5) Without a RTX control group, it is not possible to directly compare the efficacy of the two CD20 monoclonal antibodies.

Future research directions: (1) Conduct a multicenter prospective randomized controlled trial (OFA vs. RTX) to clarify the relative efficacy and safety advantages of OFA; (2) Explore personalized dosing strategies, such as adjusting injection intervals based on B-cell kinetics or employing shorter intensive regimens for severe cases; (3) Extend follow-up to 3–5 years, focusing on long-term recurrence rates, psychiatric sequelae, cognitive function, social functioning, and long-term infection risks, to evaluate the drug’s long-term prognostic value.

## Conclusion

In summary, OFA can be utilized in patients with refractory anti-NMDAR encephalitis who have shown poor response to first-line and maintenance immunotherapy. It rapidly and effectively improves neuropsychiatric symptoms, reduces CD20+ B cell levels, promotes absorption of intracranial lesions, and enhances cognitive function. OFA demonstrates favorable safety profiles with mild adverse reactions, and its subcutaneous administration enhances treatment convenience. Although current evidence is limited, OFA represents a promising second-line immunotherapy option for refractory anti-NMDAR encephalitis. Future large-scale, prospective, controlled studies are required to further validate its therapeutic advantages, safety profile, and optimal dosing regimen.

## Data Availability

The original contributions presented in the study are included in the article/supplementary material. Further inquiries can be directed to the corresponding authors.
